# Hepatotoxicity of Contemporary Antiretroviral Drugs: A Review and Evaluation of Published Clinical Data

**DOI:** 10.3390/cells10051263

**Published:** 2021-05-20

**Authors:** Ashley O. Otto, Christina G. Rivera, John D. Zeuli, Zelalem Temesgen

**Affiliations:** 1 Department of Pharmacy, Mayo Clinic, Rochester, MN 55905, USA; otto.ashley@mayo.edu (A.O.O.); Rivera.Christina@mayo.edu (C.G.R.); Zeuli.John@mayo.edu (J.D.Z.); 2 Division of Infectious Diseases, Mayo Clinic, Rochester, MN 55905, USA

**Keywords:** human immunodeficiency virus, hepatotoxicity, antiretroviral therapy

## Abstract

Contemporary antiretroviral agents afford enhanced potency and safety for patients living with HIV. Newer antiretroviral drugs are often better tolerated than those initially approved in the early stages of the HIV epidemic. While the safety profile has improved, adverse drug reactions still occur. We have segregated the antiretroviral agents used in contemporary practice into class groupings based on their mechanism of antiviral activity (non-nucleoside reverse transcriptase inhibitors, nucleoside reverse transcriptase inhibitors, integrase inhibitors, protease inhibitors, and entry inhibitors) while providing a review and discussion of the hepatoxicity seen in the most relevant clinical literature published to date. Clinical literature for individual agents is discussed and agent comparisons afforded within each group in tabular format. Our review will provide a summative overview of the incidence and medications associated with hepatic adverse reactions linked to the use of contemporary antiretroviral drugs.

## 1. Introduction

Since the introduction into practice of the first antiretroviral drug zidovudine in 1987, the development of new antiretroviral drugs has evolved at a rapid pace. The Food and Drug Administration (FDA) has approved 34 antiretroviral drugs (characterized by eight different mechanisms of antiviral activity) and 24 fixed-dose combinations for the treatment of the HIV infection [1]. Antiretroviral therapy itself has evolved from regimens with high pill burden, an inconvenient multiple daily dosing schedule, and treatment-limiting toxicities, to the current era of fixed-dose combinations and single-tablet regimens, allowing the entire treatment to be provided with a once-daily single tablet. Furthermore, dual-drug and long-acting injectable therapies have entered clinical practice [2,3]. Antiretroviral drugs introduced in recent years are more potent and much better tolerated than their earlier counterparts. However, their use is not devoid of adverse drug reactions; these continue to be encountered, albeit at a lower rate than with older antiretroviral drugs.

As the organ primarily responsible for the metabolism of many medications, the liver is a common target for drug-induced injury. This holds true for antiretroviral drugs [4,5]. In this article, we provide a review of the liver-adverse drug reactions associated with the antiretroviral drugs actively used in the contemporary treatment of the HIV infection (Table 1). Older drugs in the antiretroviral classes of nucleoside/nucleotide reverse transcriptase inhibitors (NRTIs), non-nucleoside reverse transcriptase inhibitors (NNRTIs), and protease inhibitors (PIs), as well as the fusion inhibitor enfuvirtide, have intentionally been left out of this review. Data for this review were obtained from papers published in the English language identified by searches of Medline that reported on the results of clinical trials of individual antiretroviral drugs, as well as case reports and case series on the hepatotoxicity of the drugs of interest. The review also includes available post-marketing safety data for the antiretroviral drugs of interest.

## 2. Non-Nucleoside Reverse Transcriptase Inhibitors

Non-nucleoside reverse transcriptase inhibitors have been historically associated with hepatic injury and toxicity [6]. Multiple mechanisms for the cause of hepatotoxicity with NNRTI use have been suggested including direct cholestatic injury, hypersensitivity reaction, or mediation of immune reconstitution syndrome, though hypersensitivity appears to be the most commonly reported cause in the literature among NNRTIs [7,8,9]. These hypersensitivity reactions are likely secondary to an intermediate metabolite created during metabolism via the cytochrome P450 pathway, leading to an immunogenic reaction [9]. A review of the clinical trials evaluating hepatic toxicity with NNRTI use can be found in Table 2.

### 2.1. Efavirenz

In a prospective study of the incidence of severe hepatotoxicity among patients receiving nevirapine-based (*n* = 256) and efavirenz-based (*n* = 312) antiretroviral therapy, grade 3 or 4 hepatotoxicity was seen more frequently in patients receiving nevirapine (15.6% vs. 8%; RR 1.9; 95% CI, 1.2–3.1). This risk was most commonly seen among individuals with chronic viral hepatitis (69%) and those prescribed protease inhibitors (82%) [10]. Similarly, the presence of grade 3 or 4 hepatotoxicity with efavirenz use was 4.5% in the 2NN trial, a randomized open-label comparison of efavirenz and nevirapine, with 5.6% and 11.1% of patients being co-infected with the hepatitis B or hepatitis C virus, respectively [11]. These data, in combination with small case reports, suggest that there is a risk of hepatotoxicity with the use of efavirenz, although less so than nevirapine [8,19].

### 2.2. Etravirine

The frequency of etravirine-associated hepatotoxicity is low [12,20]. In “Demonstrate undetectable viral load in patients experienced with ARV therapy” (DUET-1 and DUET-2), phase 3 clinical trials on the safety and efficacy of etravirine in antiretroviral treatment-experienced patients, found that the rate of hepatic adverse effects was not statistically significantly different between etravirine and placebo (8.7% vs. 7.1%; 95% CI −1.5–4.6; *p* = 0.3370). The incidence of grade 3 or 4 increases in alanine transaminase (ALT; 4.4% vs. 2.3%; *p* = 0.0540) or aspartate aminotransferase (AST; 3.9% vs. 2.5%; *p* = 0.1899) were minimal between etravirine and a placebo, respectively. Concomitant hepatitis C virus and etravirine use did not appear to increase the risk of hepatotoxicity [21].

### 2.3. Rilpivirine

Rilpivirine use, in comparison to efavirenz, is associated with fewer liver-related adverse effects, and appears to occur more frequently in individuals co-infected with either the hepatitis B or hepatitis C virus [14,15,22]. The “Efficacy comparison in treatment-naive, HIV-infected subjects of TMC278 and efavirenz” (ECHO) and “TMC278 against HIV, in a once-daily regimen versus efavirenz” (THRIVE) trials were phase 3, non-inferiority trials comparing rilpivirine versus efavirenz in combination with two NNRTIs. In the ECHO trial, grade 3 or 4 elevations in ALT (1%; 4/345 rilpivirine vs. 4%; 12/340 efavirenz) and AST (2%; 8/345 rilpivirine vs. 4%; 12/339 efavirenz) were less common in the rilpivirine arm versus efavirenz [13]. Similarly, in the THRIVE trial, grade 3 or 4 elevations in ALT (2%; 6/340 rilpivirine vs. 3%; 11/330 efavirenz) and AST (2%; 6/340 rilpivirine vs. 4%; 7/330 efavirenz) were less common with rilpivirine [14]. Using pooled data from the ECHO and THRIVE trials, Nelson and colleagues evaluated rilpivirine-based therapy in those with HIV and concomitant hepatitis B and/or hepatitis C infection (N = 686) versus those who received efavirenz-based therapy. Hepatic adverse effects possibly related to drug treatment were seen in 2.2% (15/686) of those who received rilpivirine versus 2.1% (14/682) in the efavirenz group, with a majority of these events being asymptomatic grade 1 or 2 increases in transaminase levels. While infrequent, hepatic adverse events requiring discontinuation of therapy occurred in 0.4% (3/682) of those receiving rilpivirine and 1.3% (9/682) of those receiving efavirenz [15].

A single-patient case report noted a 27-year-old male patient with treatment-experienced HIV-1 infection who transitioned from raltegravir plus abacavir/lamivudine to rilpivirine plus abacavir/lamivudine. Fourteen weeks after the switch, the patient’s ALT was 231 IU/L and total bilirubin was 31 µmol/L. Other causes of liver injury were explored and were all negative or normal. Liver histology revealed confluent centrilobular necrosis suggesting possible drug-induced liver injury. Resolution of ALT and total bilirubin were seen with the transition back to raltegravir plus abacavir/lamivudine [23].

### 2.4. Doravirine

Doravirine appears to be infrequently associated with transient elevations in transaminases and has not been implicated in cases of acute hepatic failure. In a randomized, dose-escalation, short-term monotherapy study of doravirine in treatment-naive HIV-1 infected patients, 1/18 (5.6%) patient developed elevated liver enzymes 24 h following the last dose of doravirine, though study authors concluded that the elevations were concurrent with a newly acquired hepatitis C diagnosis [24]. In landmark trials, “Doravirine versus ritonavir-boosted darunavir in antiretroviral-naïve adults with HIV-1” (DRIVE-FORWARD), “Doravirine/lamivudine/tenofovir disoproxil fumarate versus efavirenz/emtricitabine/tenofovir disoproxil fumarate in treatment-naïve adults with HIV-1 infection” (DRIVE-AHEAD), and “Switching to doravirine/lamivudine/tenofovir disoproxil fumarate maintains HIV-1 virologic suppression” (DRIVE-SHIFT), ALT elevations above five times the upper limit of normal (ULN) occurred in less than 2% of patients enrolled and did not require medication discontinuation [16,17,18]. Grade 2 bilirubin elevations were seen in 7/383 (2%) patients who received doravirine, though these were transient and patients did not require antiretroviral discontinuation [16]. At the time of writing, there are no published case reports or post-marketing data that associate doravirine with liver injury.

## 3. Nucleoside Reverse Transcriptase Inhibitors

Nucleoside reverse transcriptase inhibitors (NRTIs) have always been important components of antiretroviral drug regimens. The hepatotoxicity associated with NRTIs may be due to mitochondrial toxicity, hypersensitivity, or flares of hepatitis. Mitochondrial toxicity occurs from inhibition of mitochondrial DNA polymerase γ (Pol γ), leading to subsequent fatty acid accumulation and an increase in pyruvate metabolism to lactate [8,25]. Older NRTIs, such as didanosine, stavudine, and zidovudine, are associated with higher rates of hepatotoxicity in comparison to more contemporary NRTIs [25]. Table 3 describes the literature surrounding the hepatic toxicity incidence of NRTI use.

### 3.1. Abacavir

Abacavir has been associated with a potentially life-threatening hypersensitivity reaction with a reported incidence of 4–6% that typically occurs within the first 2–6 weeks of use [32]. Abacavir hypersensitivity reaction has been associated with a genetic predisposition, HLA B*5701, and can result in minor elevations in transaminase levels. However, there have been reports describing abacavir-associated liver injury in the setting of negative HLA B*5701 and hepatitis B/C testing. In all reported cases, cessation of abacavir led to improvement or normalization of transaminase levels [27,28,33].

### 3.2. Emtricitabine and Lamivudine

Emtricitabine has demonstrated little evidence of direct hepatotoxicity. This may be due to the minimal hepatic metabolism that emtricitabine undergoes or its chemical structure that inhibits strong binding to Pol γ [8,32,33]. Emtricitabine is active against the hepatitis B virus (HBV). Patients with chronic HBV may experience hepatitis flares when started on emtricitabine due to immune reconstitution secondary to dramatic shifts in viral replication [33,34,35,36]. Patients with HBV on emtricitabine may also experience post-treatment exacerbations of HBV infection on discontinuation. This mechanism of post-treatment exacerbation is hypothesized to be secondary to cytotoxic T cell recognition of viral peptides and binding to TNF ligands on inflammatory cells. In an evaluation of long-term studies of emtricitabine monotherapy in HBV treatment, the incidence of post-treatment exacerbations ranged from 7% with short-term treatment, to 23% with a median time to onset of around 11 months [37].

Toxicity with lamivudine use occurs infrequently, similarly to that of emtricitabine, given the minimal hepatic metabolism and weaker binding to Pol γ and is likely primarily associated with hepatitis flares as described above [32]. Three case reports published describe hepatic decompensation with lamivudine. The first case described a patient coinfected with HIV and HBV who developed hepatotoxicity with a combination of lamivudine and stavudine possibly secondary to drug toxicity versus reactivation of HBV [29]. In a second case, a coinfected patient developed hepatic necrosis with a combination of lamivudine/zidovudine/indinavir, with lamivudine re-initiation after recovery [38]. A third case described a patient with chronic HBV initiated on lamivudine who developed hepatic failure requiring liver transplantation, possibly due to drug-induced toxicity versus hepatitis flare [30]. While infrequent, lamivudine use may cause elevations in liver transaminases with the possibility of severe hepatotoxic effects.

### 3.3. Tenofovir

Similar to emtricitabine and lamivudine, tenofovir may cause transient elevations during or after therapy, especially when used in the management of HBV due to treatment or withdrawal flares. In reviewing data on tenofovir disoproxil fumarate use in pre-exposure prophylaxis, mild increases in liver transaminase levels are seen, but rarely (<1%) do individuals develop hepatotoxicity defined as transaminases ≥5 times ULN [32,39,40]. Tenofovir disoproxil fumarate and tenofovir alafenamide increase the concentrations of other concomitant antiretrovirals, such as efavirenz or didanosine, predisposing patients to elevated transaminase levels or mitochondrial toxicity [41,42,43]. The “Emtricitabine and tenofovir alafenamide versus emtricitabine and tenofovir disoproxil fumarate for HIV pre-exposure prophylaxis” (DISCOVER) study, a phase 3 pre-exposure prophylaxis (PrEP) trial comparing tenofovir alafenamide and tenofovir disoproxil fumarate (in combination with emtricitabine), reported grade 3/4 AST/ALT elevations at 2% in both groups [31].

## 4. Integrase Strand Transfer Inhibitors

Integrase strand transfer inhibitors (INSTIs) have emerged as key components of initial antiretroviral regimens given their virologic efficacy and tolerability. Hepatotoxicity associated with INSTIs is rarely reported in the literature with no describing mechanism listed for when it does occur (Table 4) [44]. In a review of the incidence of hepatotoxicity with INSTI use in 4366 people participating in The EuroSIDA study, a prospective observational pan-European cohort study of people living with HIV-1 across Europe, there was only one discontinuation due to hepatotoxicity [45].

### 4.1. Raltegravir

Raltegravir was the first INSTI available on the market. Phase III clinical trials “Blocking integrase in treatment experienced patients with a novel compound against HIV: Merck” (BENCHMRK-1 and BENCHMRK-2) and “Long-term treatment with raltegravir or efavirenz combined with tenofovir/emtricitabine for treatment-naive human immunodeficiency virus-1-infected patients” (STARTMRK) describe an evaluation of hepatic events. BENCHMRK-1 and BENCHMRK-2 were randomized, placebo-controlled, phase 3 trials that investigated the efficacy and safety of raltegravir plus an optimized background regimen in previously treated patients with multidrug-resistant HIV-1. STARTMRK was a phase 3 study of antiretroviral treatment-naive patients that compared the efficacy of raltegravir to efavirenz, each used in combination with tenofovir/emtricitabine. All three studies excluded individuals with cirrhosis given raltegravir’s primary hepatic metabolism. In BENCHMRK I and II, hepatic adverse events were similar between both groups [46,59]. One patient in the raltegravir arm required therapy discontinuation secondary to elevations in transaminases, though this was deemed not to be drug-related [60]. Similar results were seen in the STARTMRK trial [47,61,62]. Subgroup analyses for all three clinical trials have been completed, with a small portion of patients with hepatitis co-infection (6% treatment-naive and 16% treatment-experienced). Grade 3 or 4 elevations in transaminases were more common in individuals who were co-infected versus those with HIV alone (3% vs. 4%), though there were no differences seen between raltegravir and the control arms [63,64]. In a case-series review, eight patients with moderate hepatic insufficiency (Child-Pugh score of 7–9) received raltegravir with no hepatotoxic events [65].

### 4.2. Elvitegravir/Cobicistat

An evaluation of elvitegravir’s implications on hepatotoxicity is difficult given the required co-administration with cobicistat. However, a single, phase IIa study evaluated 10 days of elvitegravir monotherapy in 40 patients; none developed hepatic adverse events [66]. In two Phase III trials, transaminase elevations occurred with a fixed-dose combination tablet containing elvitegravir. The GS-236-0103 study compared elvitegravir/cobicistat/emtricitabine/tenofovir disoproxil fumarate versus atazanavir/ritonavir+ emtricitabine/tenofovir disoproxil fumarate for initial treatment of HIV-1 infection and reported elevations in ALT of any grade (21.6% vs. 15.3%, respectively) [48]. Similarly, GS-US-236-0102 compared elvitegravir/cobicistat/emtricitabine/tenofovir disoproxil fumarate versus efavirenz/emtricitabine/tenofovir disoproxil fumarate for initial treatment of HIV-1 infection; elevations in transaminases were more frequent in the efavirenz group: ALT (18% vs. 31%, respectively) and AST (15% vs. 35%, respectively) [49]. For patients co-infected with HIV and hepatitis B virus, normalization of ALT levels was observed in approximately 50% of patients in the elvitegravir/cobicistat arm who had elevated baseline ALT levels, though this was primarily in individuals with suppressed hepatitis B virus DNA [67]. In the “Surveillance cohort long-term antiretrovirals in HIV-infected patients enrolled in TPV cohort” (SCOLTA) project, a multicenter, observational study reporting adverse events in subjects initiating elvitegravir/cobicistat/emtricitabine/tenofovir disoproxil fumarate, grade 1–2 transaminase elevations were noted in 17/202 (8.5%) treatment-experienced patients and 3/78 (3.8%) treatment-naive subjects. Similarly, grade 3–4 transaminase elevations were noted in 2/202 (1%) treatment-experienced patients and 1/78 (1.3%) treatment-naive subjects. HCV-RNA-positive subjects had a significantly higher proportion of liver-related adverse events [50].

### 4.3. Dolutegravir

Primary data on dolutegravir monotherapy notes no hepatic adverse effects in a 10-day trial [51]. In the “Once daily dolutegravir (S/GSK1349572) in combination therapy in antiretroviral-naive adults with HIV“ (SPRING-1) trial, a phase IIb, dose-ranging study of dolutegravir in treatment-naive individuals, one patient receiving dolutegravir developed a grade 3 or 4 elevation in AST, though not related to dolutegravir use per the authors [52]. In the “Once-daily dolutegravir versus raltegravir in antiretroviral-naive adults with HIV-1 infection” (SPRING-2) trial, a phase 3 clinical trial that compared the efficacy and safety of dolutegravir versus raltegravir as first-line treatment for antiretroviral-naive adults, two patients receiving dolutegravir + emtricitabine/tenofovir disoproxil fumarate or abacavir/lamivudine developed increases in ALT at least five times ULN, requiring discontinuation, with one of those patients possibly developing a dolutegravir-induced liver injury with associated hypersensitivity [53]. There have been case reports of patients on dolutegravir/abacavir/lamivudine presenting with liver injury, with liver biopsies suggesting mitochondrial toxicity [68,69].

### 4.4. Bictegravir

As a newer integrase inhibitor, data on hepatic complications associated with bictegravir are limited. Primary insights come from Phase II and III data. In a Phase II trial comparing the safety and efficacy of bictegravir/emtricitabine/tenofovir alafenamide versus dolutegravir/emtricitabine/tenofovir alafenamide, 6/64 patients (9%) in the bictegravir arm developed grade 2–4 elevations in AST versus 1/32 patient (3%) in the dolutegravir arm; 4/64 patients (6%) in the bictegravir arm versus no patients in the dolutegravir arm developed ALT elevations. One of the patients in the bictegravir arm was diagnosed with hepatitis C coinfection with active alcohol use. All other elevations were transient and resolved even when therapy was continued [54]. Week 144 data of the two Phase III clinical trials noted non-inferiority of bictegravir/emtricitabine/tenofovir alafenamide versus dolutegravir/abacavir/lamivudine (Study 1489) or dolutegravir/emtricitabine/tenofovir alafenamide (Study 1490). In Study 1489, grade 3 or 4 elevations in ALT (2% vs. 2%) and AST (5% vs. 3%) were infrequently seen between the bictegravir-based regimen versus the dolutegravir-based regimens [55]. This was similarly seen in Study 1490 for ALT (3% vs. 1%) and AST (2% vs. 3%) [56]. No discontinuations in therapy occurred from these elevations. For those patients co-infected with hepatitis B and HIV, there were no hepatic adverse effects or discontinuations due to hepatic outcomes [55,56]. Results from week 144 were similar to that of weeks 48 and 96 [70]. At this time, there are no case reports suggesting liver injury associated with bictegravir use.

### 4.5. Cabotegravir

Cabotegravir is the newest antiretroviral in the INSTI class. Cabotegravir oral tablets, to be taken with oral rilpivirine, are used for lead-in therapy prior to initiating cabotegravir/rilpivirine long-acting intramuscular injections [71,72]. Given the co-administration, evaluating the individual hepatotoxic risk of cabotegravir is difficult. However, a phase I, single-dose study of cabotegravir 30 mg was evaluated in 16 patients with moderate hepatic impairment (Child-Pugh scores of 7–9) versus a matched healthy cohort. One patient in the hepatic impairment group developed grade 3 elevations in direct bilirubin; however, this was not thought to be clinically significant or reported as an adverse effect [73]. In the “Evaluate the safety tolerability and acceptability of long-acting injections of the HIV integrase inhibitor, GSK1265744, in HIV-uninfected men” (ECLAIR) trial, a phase 2a study assessing the safety, tolerability, and pharmacokinetics of long-acting cabotegravir versus a placebo in healthy men not at high risk of HIV-1 infection, one patient developed acute HIV-1 infection and had grade 3 elevations in ALT and grade 2 elevations in AST at week 53 [57]. The combination of cabotegravir/rilpivirine has been evaluated in the phase 3 “First long-acting injectable regimen” (FLAIR) and “Antiretroviral therapy as long acting suppression” (ATLAS) trials. These randomized, open-label trials compared the safety and efficacy of monthly cabotegravir long-acting plus rilpivirine long-acting to that of a conventional 3-drug oral antiretroviral regimen in treatment-experienced patients (ATLAS) and in previously treatment-naive patients (FLAIR). Week 48 pooled analyses note ALT and AST elevations greater than or equal to 5 times ULN in 2% of patients versus <1% in the prior ART regimen. Minor increases in total bilirubin, without jaundice, were noted in the cabotegravir/rilpivirine groups. This was described as non-clinically significant and thought to be secondary to cabotegravir and unconjugated bilirubin competition for clearance via UGT1A1 [58].

## 5. Protease Inhibitors

Protease inhibitors (PIs) are an integral part of HIV treatment, particularly for those who are treatment-experienced. PIs in contemporary use (atazanavir, darunavir, lopinavir) are paired with low-dose ritonavir or cobicistat as pharmacologic boosters [74]. As a drug class, PIs are associated with adverse effects including dyslipidemia, hepatotoxicity, and lipodystrophy [75]. PIs carry warnings for increased ALT/AST in those with viral hepatitis or pre-existing liver disease, acute hepatitis leading to hepatic failure and death. However, attribution of hepatic toxicity to PIs alone can be challenging given common confounding factors such as drug-drug interactions, polypharmacy, comorbidities, and co-infection with hepatitis B and/or C; a defined injury mechanism for the PI class is also lacking [76]. Table 5 describes a literature review of the incidence and evaluation of hepatotoxicity associated with PI use.

### 5.1. Atazanavir Sulfate

Atazanavir is well known to cause a reversible increase in serum unconjugated bilirubin without inducing liver injury; this is secondary to UGT1A1 enzyme inhibition, analogous to a chemical induction of Gilbert’s Syndrome [84]. The prevalence and risk factors for atazanavir/ritonavir-associated hyperbilirubinemia were analyzed using data from the Italian atazanavir expanded access use program and the Italian Management Standardizzato di Terapia antiretrovirale (MASTER) cohort, a hospital-based multicenter, observational study. In the HIV-infected cohort, grade III hyperbilirubinemia was observed in 1072 (44.6%) and grade IV in 174 (7.2%) patients. The risk factors for grade ≥ III hyperbilirubinemia were higher CD4+ T-cell counts, abnormal bilirubinemia at baseline, and ritonavir co-administration. The occurrence of grade ≥ III hyperbilirubinemia was not associated with severe hepatotoxicity (hazard ratio 1.00, 95% confidence interval 0.64–1.57; *p* = 0.997). Hyperbilirubinemia associated with atazanavir/ritonavir was considered to be common and not a cause of significant hepatotoxicity [77]. The findings echoed those of atazanavir registrational trials, where incidence of liver injury, defined as increases in ALT or AST > 5 times ULN, was low at 2–7 cases per 100 patients; the frequency of increases in total bilirubin > 2.5 times ULN was higher at 22–47 cases per 100 patients [85].

The impact of hyperbilirubinemia on atazanavir/ritonavir discontinuation rates, quality of life, and drug adherence was examined in the “Once-daily atazanavir/ritonavir versus twice-daily lopinavir/ritonavir, each in combination with tenofovir and emtricitabine, for management of antiretroviral-naïve HIV-1-infected patients” (CASTLE) study, a randomized study that compared atazanavir/ritonavir to lopinavir/ritonavir in treatment-naïve HIV-infected patients. Forty-four percent of patients receiving atazanavir/ritonavir experienced hyperbilirubinemia, 5% experienced jaundice (11% of those with hyperbilirubinemia), and 7% of all patients had grade 3–4 LFT elevations (4% of those with hyperbilirubinemia). Less than 1% of patients discontinued treatment due to hyperbilirubinemia. Quality of life and adherence were the same in those with and without hyperbilirubinemia. The investigators concluded that hyperbilirubinemia did not negatively impact clinical outcomes in HIV-infected patients taking atazanavir/ritonavir [78].

Atazanavir co-formulated with cobicistat also carries a warning for hyperbilirubinemia [86]. In a phase 3 clinical trial comparing atazanavir plus cobicistat versus atazanavir/ritonavir, both in combination with emtricitabine/tenofovir disoproxil fumarate (FTC/TDF), the proportion of patients experiencing jaundice was higher in the atazanavir/cobicistat arm (6% jaundice, 4% scleral icterus) than in those receiving atazanavir/ritonavir (3% jaundice, 4% scleral icterus). However, odds ratios for drug discontinuation due to adverse events did not significantly differ between the regimens overall at weeks 48 and 144 (OR 0.98; 95% CI: 0.61, 1.58) [79,86].

Atazanavir-induced hyperbilirubinemia occurring during pregnancy requires special consideration. An observational study of 22 HIV-infected women receiving atazanavir/ritonavir during pregnancy and their 23 infants revealed median cord blood atazanavir concentration was 130 ng/mL (range < 30–758) with a cord/maternal ratio of 21%. Bilirubin concentrations at birth were significantly higher than maternal concentrations, with a median of 44 μm/L (range 24–129); values on days 2–3 were 63 μm/L (range 8–212). Three neonates had mildly elevated AST levels. Five neonates had jaundice requiring phototherapy but did not experience liver damage [87].While all babies in this study recovered without short-term sequelae, the potential for negative effects on neonatal neurodevelopment from in utero hyperbilirubinemia from atazanavir/ritonavir exposure remains a concern [88].

### 5.2. Lopinavir/Ritonavir

In clinical trials, lopinavir/ritonavir was associated with a 2–9% incidence of hepatotoxicity with the concomitant presence of HCV infection, imparting a 4.7-fold increase in LFT abnormalities [80,89]. A retrospective analysis of 120 patients living with HIV, of liver toxicity incidence after initiation of lopinavir and possible correlation with lopinavir plasma levels, found that severe liver toxicity occurred in 1.7% of subjects at three months with a cumulative incidence at 12 months of 4%, and confirmed an association with HCV co-infection but not with lopinavir plasma levels [90]. These data were confirmed in an observational, comparative, prospective study of 78 (HIV-positive/HCV-negative) and 71 (HIV-positive/HCV-positive) non-cirrhotic patients receiving lopinavir/ritonavir. Increases in transaminases were significantly higher in co-infected (HIV-positive/HCV-positive) subjects and did not correlate with lopinavir trough concentrations [91]. Despite the higher risk of hepatotoxicity in those with HCV coinfection, the presence of hepatitis B or C is not considered a contraindication to lopinavir/ritonavir use [74].

### 5.3. Darunavir

In the “Performance of TMC114/r when evaluated in treatment-experienced patients with PI resistance” (POWER-1 and POWER-2) trials, randomized, phase IIB studies of the efficacy and safety of darunavir in combination with low-dose ritonavir in treatment-experienced HIV-1-infected patients, darunavir/ritonavir was associated with moderate-to-severe LFT elevations in 3–10% of patients. The liver injury occurred generally at one to eight weeks following initiation of treatment, usually in a hepatocellular pattern with the absence of chronic hepatitis [92]. In an analysis of data from the “Italian cohort of individuals, naïve for antiretrovirals” (ICONA) Foundation Cohort, 703 patients, of which 68 (9.7%) had active HCV coinfection, were assessed for the rate of liver enzyme elevation and severe hepatotoxicity with the initiation of darunavir/ritonavir. HCV-coinfected patients experienced low-grade liver enzyme elevations more frequently than HCV-antibody-negative patients; no grade 3–4 liver enzyme elevations were observed [93]. A case report highlighted darunavir/ritonavir as a cause of cholestatic hepatitis three years after initiating antiretroviral therapy that resolved only after changing darunavir/ritonavir to an INSTI [94]. Ongoing liver function monitoring in patients receiving darunavir/ritonavir is indicated and occurrence of significant liver enzyme elevations should at a minimum prompt consideration of darunavir/ritonavir involvement and possibly discontinuation. Largely based on the darunavir/ritonavir experience, darunavir co-formulated with cobicistat carries a similar recommendation to consider increased AST/ALT monitoring in patients with underlying chronic hepatitis, cirrhosis, or in patients who have pre-treatment elevations of transaminases, particularly during the first several months of therapy. Darunavir should be discontinued with progression of liver injury [95].

## 6. Entry Inhibitors

### 6.1. Maraviroc

Maraviroc selectively binds to the human chemokine CCR5 receptor, blocking the necessary interaction of GP120 and CCR5 for viral fusion and entry into CD4 cells. Maraviroc received FDA approval in August 2007 for use for treatment-experienced patients and carries a black box warning for hepatotoxicity. However, the combined clinical trial data and extended evaluation of maraviroc use over five years in close to 1000 patients do not justify the concern prompted by the black box warning [96].

During early clinical development of maraviroc, a study patient experienced acute hepatocellular injury with rash, fever, and eosinophilia, which was attributed to maraviroc. This occurred shortly after clinical development of aplaviroc (another CCR5 inhibitor) was terminated in 2005 due to unacceptable hepatoxicity [97]. The mechanism for aplaviroc toxicity appeared to be idiosyncratic drug toxicity leading to cytolysis (potentially with association of an unknown cofactor) [98]. Heightened concerns of liver damage as a potential class effect of CCR5 inhibitors prompted the FDA to require inclusion of a black box warning on the label. The FDA wanted to heighten provider awareness of potential liver damage during manufacturer promotion of maraviroc, given that maraviroc was the first agent approved in a new class of antiretroviral therapy (CCR5 inhibitors) [99].

Safety data from 2350 patients during clinical development show maraviroc has a low incidence of associated liver toxicity through phase 1/2a trials and up to 96 weeks of phase 2b/3 evaluation in both treatment-naïve and treatment-experienced patients [100]. Healthy volunteers in phase 1 multiple-dose studies did not show any hyperbilirubinemia >2.5× ULN, and only a few events of transaminase elevation occurred without any correlation to dose (Table 6) [100].

The “Maraviroc versus efavirenz in treatment-naive patients” (MERIT) study evaluated maraviroc twice daily versus efavirenz, each combined with co-formulated zidovudine/lamivudine, in treatment-naïve patients with CCR5-tropic (R5) HIV-1. Comparable drug exposure occurred between groups (506.0 and 507.9 patient years, respectively) through 96 weeks. No significant differences between grade 1/2, grade 3, or grade 4 elevations of ALT were seen, and equivalent proportions of patients (24.9% vs. 23.1%) had an increase of one grade from the baseline during the study (Table 7). No bilirubin-related grade 4 lab abnormalities occurred and only three grade 3 abnormalities were observed (two attributable to Gilbert’s syndrome). None of the grade 3 events corresponded with increased transaminases. Only one patient discontinued maraviroc due to a drug-related hepatobiliary event. One patient in the maraviroc once daily arm of MERIT developed hepatic failure requiring a transplant; this occurred after the patient discontinued maraviroc and in the setting of concomitant isoniazid, trimethoprim/sulfamethoxazole, lopinavir-ritonavir, and acetaminophen exposure. These other drugs were deemed likely causes of the liver failure, though maraviroc could not be excluded [101,102].

“Maraviroc therapy in antiretroviral treatment-experienced HIV-1 infected patients” (MOTIVATE 1 and 2) evaluated maraviroc versus a placebo in combination with an optimized background regimen through 96 weeks in a pair of phase 3 studies of treatment-experienced patients [103]. Patients with transaminase levels >5× ULN or bilirubin >2.5× ULN at the baseline were excluded from the MOTIVATE trials, but patients coinfected with HBV and HCV could enroll provided they did not exhibit baseline liver exclusion criteria. ALT elevation event rates in the trials were normalized for time due to the shorter duration of optimized background regimen (OBT) on account of more regimen failure in this arm. 

Event rates from MOTIVATE 1 and 2 are provided in Table 8 [104]. Grade 3 and 4 ALT event rates were lower in both maraviroc arms compared to a placebo. Overall treatment-related hepatobiliary adverse effects were low and not significantly different between treatment arms, as were discontinuations due to hepatobiliary AEs. Given the previously discussed concerns for hepatoxicity of maraviroc upon approval, the FDA requested a five-year follow-up for all study subjects in the MOTIVATE trials. This evaluation assessed death and clinical safety endpoints (to include hepatic failure). Overall rates were very low, and maraviroc was concluded to be generally safe from the review of the 938 evaluable patients with 2639 patient years of exposure. Only five events (0.5%) of hepatic failure were seen during this evaluation period [96,105]. Additionally, as of 12/31/2020, the FDA Adverse Events Reporting System lists 146 post-marketing reported cases of hepatobiliary disorders (including 15 cases of death) out of a total of 1731 reports [106]. This ranks eighth behind other reported adverse effects with maraviroc to include infections, nervous system disorders, gastrointestinal disorders, and cardiac disorders. Notwithstanding the limitations of post-marketing public reporting, the low relative signal of hepatobiliary complications would support low hepatotoxicity rates described in clinical development.

Hepatitis B and C coinfection rates varied between approximately 4–8% in the MERIT and MOTIVATE study arms, but co-infection during the study timeframe did not appear to affect the hepatobiliary adverse effect incidence in the study populations, nor did it impact differences between groups.

In summary, the initial concerns of hepatoxicity of maraviroc have not been supported. Extensive clinical data demonstrate safe and successful use of maraviroc through 2300 clinical trials participants, 96-week safety results from the MOTIVATE and MERIT study populations, a five-year planned safety analysis, and lack of a significant signal in post-marketing reports.

### 6.2. Ibalizumab

Ibalizumab-uiyk is a recombinant humanized monoclonal antibody. It exerts an antiviral effect by binding to domain 2 of the CD4 receptor. When the HIV GP120 protein binds to the CD4 receptor, steric hindrance from ibalizumab prevents the conformational changes necessary for fusion and viral entry into the cell. 

Clearance of ibalizumab occurs via protein and cellular degradation [107]. Ibalizumab does not require hepatic phase 1 or 2 metabolism, nor is ibalizumab expected to concentrate in the liver, so toxic hepatic effects are not anticipated. This is reflected in the available clinical trial data to date in heavily treatment-experienced patients with advanced drug-resistant HIV infection.

In the 40 evaluable patients who received at least one dose of study drug in TMB-301 through the 24 week study period, there were no reports of grade 3/4 transaminase elevations, and no evidence of hepatoxicity attributable to ibalizumab [108,109]. There were only two cases of bilirubin elevation >2.5× ULN, neither attributable to ibalizumab. One death occurred as a result of hepatic failure; this was attributed to decompensated cirrhosis secondary to hepatitis C infection and was not deemed study drug-related. 

Safety analysis for trial extension through 96 weeks failed to identify any drug attributable hepatotoxicity or any liver safety issues [110,111]. Furthermore, a subgroup of 12 patients that started ibalizumab during the phase 2b study (TMB-202) continued drug through expanded access protocol for a mean of nine years without any demonstrated hepatoxicity or liver safety signals attributed to ibalizumab [112]. The mechanism of drug action, metabolic/pharmacokinetic profile, and summative data to date suggest that ibalizumab does not pose a hepatoxicity concern.

### 6.3. Fostemsavir

Fostemsavir is a prodrug that is hydrolyzed to the active agent, temsavir. Temsavir binds directly to GP120 and prevents attachment to CD4 receptors. 

Four dosing approaches for fostemsavir (400 mg twice daily, 800 mg twice daily, 600 mg once daily, and 1200 mg once daily) were all well tolerated in 200 patients through 48 weeks in AI438011, a phase 2 clinical trial that compared the safety and efficacy of fostemsavir vs. ritonavir-boosted atazanavir (each in combination with raltegravir and tenofovir DF) in treatment-experienced HIV-1-infected subjects. No discontinuations due to drug-related hepatic adverse effects occurred [113]. At 48 weeks, patients all transitioned to the fostemsavir 1200 mg once daily dosing scheme. Long-term follow-up of this cohort through 192 weeks (median duration of 4.5 years) yielded no discontinuations due to a hepatobiliary adverse effect, suggesting long term fostemsavir use is not associated with hepatoxicity [114].

The “Fostemsavir in adults with multidrug-resistant HIV-1 infection” (BRIGHTE) phase 3 study evaluated fostemsavir 600 mg twice daily in addition to an optimized background regimen in 371 treatment-experienced patients with HIV, stratified in two cohorts by the available number of fully active antiretroviral agents. Fostemsavir did not demonstrate significant hepatotoxic potential. The percentage of patients who increased to grade 3 or 4 laboratory abnormalities from baseline was low [115]. Only three hepatobiliary adverse events during the study period led to discontinuation: two hepatic failures and one case of hepatorenal syndrome. All three events were attributed to underlying disease and not fostemsavir.

Twenty-five deaths (7%) occurred during the trial, mostly attributable to underlying disease and/or opportunistic illness (of those who died, their mean CD4 was 11). Two of the deaths were caused by hepatic failure (one resulting from a flare of hepatitis B and the other from progression of hepatitis C) and, again, not related to fostemsavir [115]. Only one death, a case of severe immune reconstitution inflammatory syndrome, was attributed to fostemsavir. Extended safety evaluation through 96 weeks in this study population did not identify any fostemsavir-related hepatobiliary complications [116].

The extended evaluation of current clinical trial results supports that fostemsavir has a low risk of contributing to hepatobiliary toxicity.

## 7. Summary and Conclusions

The antiretroviral drugs used in the contemporary treatment of HIV infection are potent and well-tolerated. However, liver-related adverse drug reactions continue to be reported, albeit at lower rates than noted with earlier drugs. There is no established standard of care for hepatic injury secondary to ART. Elimination and/or minimization of other hepatotoxins (i.e., acetaminophen, alcohol) is a sensible first step. Screening for and treating viral hepatitis as indicated is also an important measure. A careful consideration of the risks and benefits of stopping or changing the suspected offending drug(s) in an ART regimen should be undertaken with the advisement of an HIV specialist. 

Monitoring patients on ART for the emergence of liver injury, in particular in those with conditions that pose a higher risk, such as viral hepatitis and alcohol use, should remain a key component of the management of HIV infection.

## Figures and Tables

**Table 1:** Antiretroviral agents (by mechanism of action) used in contemporary management of HIV.

Nucleoside/Nucleotide Reverse Transcriptase Inhibitors (NRTIs)	Non-Nucleoside Reverse Transcriptase Inhibitors (NNRTIs)	Protease Inhibitors (PIs)	Integrase Strand Transfer Inhibitors (INSTIs)	CCR5 Antagonist	CD4-Directed Post-Attachment Inhibitor	Attachment Inhibitor
Abacavir (ABC)	Doravirine (DOR)	Atazanavir (ATV)	Raltegravir (RAL)	Maraviroc (MVC)	Ibalizumab (IBA)	Fostemsavir (FTR)
Emtricitabine (FTC)	Efavirenz (EFV)	Darunavir (DRV)	Elvitegravir (EVG)			
Lamivudine (3TC)	Etravirine (ETR)	Lopinavir (LPV)	Dolutegravir (DTG)			
Tenofovir disoproxil fumarate (TDF)	Rilpivirine (RPV)		Bictegravir (BIC)			
Tenofovir alafenamide (TAF)			Cabotegravir (CAB)			

**Table 2:** Clinical trial evaluation of hepatic toxicity and incidence for non-nucleoside reverse transcriptase inhibitors.

Reference	Drug(s)	No. of Study Patients	Hepatic Evaluation	Overall Incidence of Cases/100 Persons Exposed	Study Design	Patient Population
Sulkowski 2002 [10]	Efavirenz	312	Combined Grade 3 and 4 Grade 3: AST/ALT 5.1–10× ULN Grade 4: AST/ALT > 10× ULN	8	Prospective	Treatment-naive; 40% HCV-positive; 52% concurrent protease inhibitor use
van Leth 2004 2NN [11]	Efavirenz	400	Combined Grade 3 and 4 Grade 3: AST/ALT 5.1–10× ULN Grade 4: AST/ALT > 10× ULN	4.5	Prospective	Treatment-naive; 10% HCV-positive; 4% HBV-positive
Girard 2012 DUET-1 and DUET 2 (96 Week Pooled Data) [12]	Etravirine	599	Grade 3: AST/ALT 5.1–10× ULN Grade 4: AST/ALT > 10× ULN	Grade 3: 4.4 Grade 4: 3.9	Prospective	Treatment-experienced; 12% HBV- and/or HCV-positive
Molina 2011 ECHO [13]	Rilpivirine	346	Combined Grade 3 and 4 Grade 3: AST/ALT 5.1–10× ULN Grade 4: AST/ALT > 10× ULN	AST: 2 ALT:1	Prospective	Treatment-naive; 3% HBV-positive; 2% HCV-positive
Cohen 2011 THRIVE [14]	Rilpivirine	340	AST/ALT 5.1–10× ULN	2	Prospective	Treatment-naive; 4% HBV-positive; 5% HCV-positive
Nelson 2012 [15]	Rilpivirine	686	Combined Grades 1–4 Grade 1: AST/ALT 1.25–2.4× ULN Grade 2: 2.5–4.9× ULN Grade 3: 5–9.9× ULN Grade 4: ≥ 10× ULN	2.2	Prospective	Treatment-naive; 8.4% HBV- and/or HCV-positive
Molina 2020 DRIVE-FORWARD [16]	Doravirine	383	AST/ALT ≥ 5× ULN	ALT: 1 AST: 2	Prospective	Treatment-naive
Orkin 2020 DRIVE-AHEAD [17]	Doravirine	363	AST/ALT 5–9.9× ULN	ALT: 0.8 AST: 0.6	Prospective	Treatment-naive; 3% HBV- and/or HCV-positive
Johnson 2019 DRIVE-SHIFT [18]	Doravirine	447	ALT/ALT ≥ 3× ULN plus bilirubin ≥ 2× ULN and alkaline phosphatase < 2× ULN	0	Prospective	Treatment-experienced; 3% HBV- and/or HCV-positive

Abbreviations: ALT, alanine transaminase; AST, aspartate aminotransferase; HBV, hepatitis B virus; HCV, hepatitis C virus; ULN, upper limit of normal.

**Table 3:** Clinical trial evaluation of hepatic toxicity and incidence for nucleoside reverse transcriptase inhibitors.

Reference	Drug(s)	No. of Study Patients	Hepatic Evaluation	Overall Incidence of Cases/100 Persons Exposed	Study Design	Patient Population
Soni 2008 [26]	Abacavir	2	Patient 1: ALT > 10× ULN Patient 2: ALT > 10× ULN	-	Case report	Patient 1: Female; HLA B*5701 negative; baseline ALT 21 IU/L Patient 2: Female; HLA B*5701 negative; baseline ALT 10 IU/L
Di Filippo 2014 [27]	Abacavir	1	AST: < 5× ULN ALT: > 10× ULN	-	Case report	Male; HLA B*5701 negative; baseline AST 27 IU/L and ALT 85 IU/L
Pezzani 2016 [28]	Abacavir	1	AST: 5× ULN ALT: > 10× ULN	-	Case report	Female; HLA B*5701 negative; baseline AST/ALT normal
Schiano 1997 [29]	Lamivudine	1	Total bilirubin: > 10× ULN ALT: > 10× ULN	-	Case report	Male; HBV co-infection; cirrhosis
Ormseth 2001 [30]	Lamivudine	1	Total bilirubin: >10× ULN ALT: > 10× ULN	-	Case report	HBV co-infection; baseline ALT 171 IU/L, bilirubin 3.1 mg/dL
Mayer 2020 DISCOVER [31]	Tenofovir		Combined grade 3 and 4 AST grade 3: >5.00 to 10.00× ULN grade 4: >10.00× ULN ALT grade 3: >5.00 to 10.00× ULN grade 4: >10.00× ULN	AST: 2 ALT: 1	Prospective	HIV-uninfected; PrEP

Abbreviations: ALT, alanine transaminase; AST, aspartate aminotransferase; HBV, hepatitis B virus; HCV, hepatitis C virus; HLA*B, major histocompatibility complex, class I, B; PrEP, pre-exposure prophylaxis; ULN, upper limit of normal.

**Table 4:** Clinical trial evaluation of hepatic toxicity and incidence for integrase strand transfer inhibitors.

Reference	Drug(s)	No. of Study Patients	Hepatic Evaluation	Overall Incidence of Cases/100 Persons Exposed	Study Design	Patient Population
Steigbigel 2010 BENCHMRK-1 and -2 (Week 96 Pooled Data) [46]	Raltegravir	462	AST/ALT > 10× ULN	AST: 0.7 ALT: 1.3	Prospective	Treatment-experienced; multidrug resistant
Lennox 2010 STARTMRK (Week 96 Data) [47]	Raltegravir	281	AST/ALT/ALK Phos > 5× ULN TBILI > 2.5× ULN	AST: 3.2 ALT: 1.8 ALK Phos: 0 TBILI: 0.7	Prospective	Treatment-naive; 6% HBV and/or HCV
DeJesus 2012 GS-236-0103 [48]	Elvitegravir/cobicistat	352	Combination of all grades for AST/ALT elevations	AST: 17.6 ALT: 15.3	Prospective	Treatment-naive; 1% HBV; 5% HCV
Sax 2012 GS-US-236-0102 [49]	Elvitegravir/cobicistat	347	Combination of all grades for AST/ALT elevations	AST: 15 ALT: 18	Prospective	Treatment-naive; 1% HBV; 5% HCV
Squillace 2017 SCOLTA [50]	Elvitegravir/cobicistat	280	Grade 1–2: AST/ALT 1.25–2.4× ULN (if baseline WNL) or baseline (if baseline value abnormal) Grade 3–4: AST/ALT ≥2.5× ULN (if baseline WNL) or baseline (if baseline value abnormal)	Grade 1–2; treatment-naive: 3.8 Grade 1–2; treatment-experienced: 8.5 Grade 3–4; treatment-naive: 1.3 Grade 3–4; treatment-experienced: 1	Prospective	72.1% treatment-experienced; 27.9% treatment-naive; 21.8% HCV
Min 2011 [51]	Dolutegravir	28	Combination of all grades for AST/ALT elevations	0	Prospective	Treatment-experienced and treatment-naive; integrase strand transfer inhibitor-naive
van Lunzen 2012 SPRING-1 [52]	Dolutegravir	205	AST/ALT ≥ 5× ULN	0.5	Prospective	Treatment-naive; 9% HCV
Raffi 2013 SPRING-2 [53]	Dolutegravir	411	AST/ALT ≥ 5× ULN	0.5	Prospective	Treatment-naive; 2% HBV; 10% HCV
Sax 2017 [54]	Bictegravir	64	Grade 2–4: AST/ALT ≥ 2.5× ULN	AST: 9 ALT: 6	Prospective	Treatment-naive
Gallant 2017 GS-US-380-1489 [55]	Bictegravir	314	Grade 3–4: AST/ALT ≥ 5× ULN	AST: 5 ALT: 2	Prospective	Treatment-naive
Sax 2017 GS-US-380-1490 [56]	Bictegravir	314	Grade 3–4: AST/ALT ≥ 5× ULN	AST: 2 ALT: 3	Prospective	Treatment-naive; 3% HBV; 2% HCV
Markowitz 2017 ECLAIR [57]	Cabotegravir	94	Grade 2–4: AST/ALT	1	Prospective	HIV-uninfected
Rizzardini 2020 FLAIR and ATLAS (Week 48 Pooled Data) [58]	Cabotegravir	591	AST/ALT ≥ 5× ULN	2	Prospective	Treatment-experienced; 7% HCV

Abbreviations: ALT, alanine transaminase; AST, aspartate aminotransferase; HBV, hepatitis B virus; HCV, hepatitis C virus; ULN, upper limit of normal.

**Table 5:** Clinical trial evaluation of hepatic toxicity and incidence for protease inhibitors.

Reference	Drug(s)	No. of Study Patients	Hepatic Evaluation	Overall Incidence of Cases/100 Persons Exposed	Study Design	Patient Population
Torti 2009 MASTER and Italian ATV [77]	Atazanavir	2404	Grade 3–4: ALT > 5× ULN Grade 3–4 TBILI > 2.5× ULN	ALT: 6.4 TBILI: 44.6	Retrospective	Longitudinal multicenter cohort; 47.3% HCV, 7.3% HBV
McDonald 2012 CASTLE [78]	Atazanavir/ ritonavir	441	Grade 3–4: AST/ALT > 5× ULN Grade 3–4 TBILI > 2.5× ULN	AST: 3 ALT: 3 TBILI: 44	Prospective	Treatment-naive
Gallant 2017 [79]	Atazanavir/ ritonavir	348	Grade 3–4: AST/ALT > 5× ULN Grade 3–4 TBILI > 2.5× ULN GGT > 5× ULN	AST: 3 ALT: 3 TBILI: 66 GGT: 2	Prospective	Treatment-naive
	Atazanavir/ cobicistat	344		AST: 4 ALT: 4 TBILI: 73 GGT: 4		
Walmsley 2002 Study 863 [80] (M-98-863)	Lopinavir/ritonavir	326	Grade 3–4: AST/ALT > 5× ULN	AST or ALT: 4.5	Prospective	Treatment-naive
González-García 2010 Study 730 [80] (M05-730)	Lopinavir/ritonavir once daily	333	Grade 3–4: AST/ALT > 5× ULN	AST: 1 ALT: 1	Prospective	Treatment-naive
	Lopinavir/ritonavir twice daily	331		AST: 2 ALT: 1		
Pollard 2004 Study 888 [80] (M98-888)	Lopinavir/ritonavir	148	Grade 3–4: AST/ALT > 5× ULN	AST: 5 ALT: 6	Prospective	Single PI-experienced, NNRTI-naive
Zajdenverg 2010 Study 802 [80] (M06-802)	Lopinavir/ritonavir once daily	300	Grade 3–4: AST/ALT > 5× ULN	AST: 3 ALT: 2	Prospective	Treatment-experienced
	Lopinavir/ritonavir twice daily	299		AST: 2 ALT: 2		
Orkin 2013 ARTEMIS [81] Week 192	Lopinavir/ritonavir	346	Grade 2–4 AST/ALT Grade 2–4 TBILI	AST: 14.9 ALT: 15.8 TBILI: 5.5	Prospective	Treatment-naive, HCV or HBV 12.5% (DRV/r) 13.9% (LPV/r)
	Darunavir/ritonavir	343		AST: 12.9 ALT: 12.6 TBILI: 1.2		
Madruga 2007 TITAN [82]	Lopinavir/ritonavir	297	Grade 2–4 AST/ALT	AST: 9 ALT: 9	Prospective	Treatment-experienced, HCV or HBV 13% (LPV/r), 18%(DRV/r)
	Darunavir/ritonavir	298		AST: 7 ALT: 9		
Arasteh 2009 POWER-1, 2, 3 (Week 96 Pooled Data) [83]	Darunavir/ritonavir	467	Grade 2–4 AST/ALT Grade 2–4 TBILI	AST: 10 ALT: 9 TBILI: 2	Prospective	Extensive treatment- experienced

Abbreviations: ALT, alanine transaminase; AST, aspartate aminotransferase; DRV/r, darunavir/ritonavir; HBV, hepatitis B virus; HCV, hepatitis C virus; LPV/r, lopinavir/ritonavir; TBILI, total bilirubin; NNRTI, non-nucleoside reverse transcriptase inhibitor; ULN, upper limit of normal.

**Table 6:** ALT and bilirubin abnormalities noted in maraviroc phase 1 multiple-dose studies.

Phase 1 Multiple-Dose Studies [100]		
ALT	Maraviroc (*n* = 272)	Placebo (*n* = 42)
>2 to ≤ 5× ULN	8 (2.9%)	0
>5× ULN	1 (0.4%)	0
Bilirubin—Total	(*n* = 272)	(*n* = 41)
>1.25 to ≤ 2.5× ULN	3 (1.1%)	0
>2.5× ULN	0	0

Abbreviations: ALT, alanine transaminase; AST, aspartate aminotransferase; ULN, upper limit of normal.

**Table 7:** ALT/Bilirubin and hepatobiliary discontinuation related to maraviroc in MERIT.

MERIT Study 96 Week Data [102]		
	MVC 300 mg Twice Daily + AZT/3TC *n* = 353	EFZ 600 mg Daily + AZT/3TC *n* = 350
ALT: Maximum value by patient over 96 weeks		
Grade 1/2 (≥1.25 to <5× ULN)	134 (38.0%)	139 (39.7%)
Grade 3 (≥5 to <10× ULN)	11 (3.1%)	12 (3.4%)
Grade 4 (≥10× ULN)	3 (0.8%)	2 (0.6%)
Bilirubin-total: Maximum value by patient over 96 weeks		
Grade 1/2 (≥1.25 to <2.5× ULN)	47 (13.3%)	5 (1.4%)
Grade 3 (≥2.5 to <5× ULN)	3 (0.8%)	0
Grade 4 (≥5× ULN)	0	0
Discontinuation due to a treatment-related hepatobiliary AE		
	1 (0.3%)	2 (0.6%)

Abbreviations: AE, adverse event; AZT, zidovudine; MVC, maraviroc; ULN, upper limit of normal; 3TC, lamivudine.

**Table 8:** ALT/bilirubin, hepatobiliary AEs/discontinuation in MOTIVATE studies.

MOTIVATE Studies 96 Week Data [104]			
	MVC 300 mg Once Daily + OBT *n* = 408	MVC 300 mg Twice Daily + OBT *n* = 421	Placebo + OBT *n* = 207
Grade 3/4 Treatment-related hepatobiliary AE	1 (0.2%)	2 (0.5%)	1 (0.5%)
Discontinuation due to any hepatobiliary AE	2 (0.5%)	2 (0.5%)	1 (0.5%)
ALT: Events per 100 years of exposure (% incidence of maximum lab value)			
Grade 1/2 (≥1.25 to <5× ULN)	55.4 (50.2%)	54.2 (51.5%)	86.8 (50.7%)
Grade 3 (≥5 to <10× ULN)	3.5 (4.4%)	1.9 (2.4%)	5.2 (3.9%)
Grade 4 (≥10× ULN)	0.4 (0.5%)	0.7 (1.0%)	1.3 (1.0%)
Bilirubin-Total: Events per 100 years of exposure (% incidence of maximum lab value)			
Grade 1/2 (≥1.25 to <2.5× ULN)	36.4 (38.2%)	30.4 (33.3%)	56.8 (36.2%)
Grade 3 (≥2.5 to <5× ULN)	7.7 (9.1%)	4.7 (5.7%)	6.7 (4.8%)
Grade 4 (≥5× ULN)	1.4 (1.7%)	0.7 (1.0%)	1.9 (1.4%)

Abbreviations: AE, adverse event; OBT, optimized background regimen, MVC, maraviroc; ULN, upper limit of normal.

## Data Availability

All relevant data are within the manuscript.
